# Integrated presentation of ecological risk from multiple stressors

**DOI:** 10.1038/srep36004

**Published:** 2016-10-26

**Authors:** Benoit Goussen, Oliver R. Price, Cecilie Rendal, Roman Ashauer

**Affiliations:** 1 Environment Department, University of York, Heslington, York YO10 5DD, UK; 2 Safety and Environmental Assurance Centre, Colworth Science Park, Unilever, Sharnbrook, Bedfordshire, UK

## Abstract

Current environmental risk assessments (ERA) do not account explicitly for ecological factors (e.g. species composition, temperature or food availability) and multiple stressors. Assessing mixtures of chemical and ecological stressors is needed as well as accounting for variability in environmental conditions and uncertainty of data and models. Here we propose a novel probabilistic ERA framework to overcome these limitations, which focusses on visualising assessment outcomes by construct-ing and interpreting prevalence plots as a quantitative prediction of risk. Key components include environmental scenarios that integrate exposure and ecology, and ecological modelling of relevant endpoints to assess the effect of a combination of stressors. Our illustrative results demonstrate the importance of regional differences in environmental conditions and the confounding interactions of stressors. Using this framework and prevalence plots provides a risk-based approach that combines risk assessment and risk management in a meaningful way and presents a truly mechanistic alternative to the threshold approach. Even whilst research continues to improve the underlying models and data, regulators and decision makers can already use the framework and prevalence plots. The integration of multiple stressors, environmental conditions and variability makes ERA more relevant and realistic.

A challenge in current environmental risk assessment (ERA) practice is accounting explicitly for multiple stressors, ecological realism, and abiotic factors that vary accross different geographies. It is widely recognised that there is a need to consider how ecological realism can be best incorporated into prospective environmental risk assessment of chemicals, as there is a need to account for variability and uncertainty in a useful, explicit and transparent manner^1^,^2^,^3^. While these complexities are currently covered by assessment factors, next generation risk assessments have the potential to explicitly account for complex interactions based on our growing understanding of the chemical, biochemical, and ecological interactions that govern adverse effects. Chemical legislation continues to evolve and develop across the globe and the scientific community is calling on ERAs to be tailored to geographical needs and thus increase our confidence in our ability to protect local biodiversity and nature^4^,^5^,^6^. A step toward capturing more ecology in ERAs of chemicals is the use of environmental scenarios that represent key differences in ecology (e.g. species composition, food availability, predation, parasitism) and the physical/habitat differences (e.g. hydrology, temperature)^7^,^8^. The incorporation of environmental scenarios into ERA will enable meaningful interpretation and extrapolation to different geographical regions.

Another driver behind the call for more ecological realism is the acknowledgement that organisms do not live in single-stress situations. Rather they are constantly exposed to a series of different stressors, both chemical and non-chemical. Organisms and populations can respond to stress using a wide range of mechanisms from a behavioural response like a change of resting site by ectotherms according to the temperature stress, an acclimation response involving changes in enzymatic activities allowing a complete or partial recovery (re-establishment of the homeostasis), or even a genetic adaptation by natural selection of genetic variants presenting a higher metabolic efficiency^9^. Over the past 15 years, several studies have attempted to assess the effects of mixtures of chemicals^10^,^11^,^12^,^13^,^14^, mixtures of ecological factors^15^,^16^ or, mixtures of chemicals and ecological factors^17^,^18^,^19^ on organisms and populations. These studies indicate that the prediction of the environmental effects of multiple stressors is a challenge that is beginning to be addressed by the scientific community. However, little effort has yet been invested in the communication of these often very complex results in an integrated and meaningful way that is easy for risk assessors to understand and interpret.

Probabilistic approaches have been suggested as a way to account for spatial, temporal, and environmental variability and to integrate multiple stressors into more ecologically relevant ERA^20^,^21^,^22^,^23^. Plots based on these approaches can present ecotoxicological data, account for ecologically relevant factors (e.g. temperature, food availability, predation, etc), and incorporate chemical exposure data on a spatial scale. Essentially such plots can help address two related questions: “How strong is the ecological effect?” and “In how many locations will we see the effect?” Our aim is to present an integrated approach to communicate results of ERA whilst accounting for variability in ecological and environmental factors. This approach enables an integrated representation of multiple stressors (e.g. chemical stress, temperature stress, and food limitation) on a single relevant figure. The output of such an analysis can support risk management decisions by assessing the prevalence (e.g. the proportion of freshwater habitats that are affected) and the magnitude of the impact (e.g. impact on populations of single species or even, in the long-term, loss of biodiversity) to allow decision makers to assess the threshold for acceptance.

## Integration of Multiple Stressors

Risk is the integrated assessment of likelihood and severity of an undesired event. In ERA of chemicals, the undesired event depends on the chemical and risk assessment scenario, and is usually a detrimental effect on organisms, populations or ecosystems. The effect not only depends on the chemical’s toxicity and concentration at the biological target site, but also on the characteristics of the organism, population or ecosystem of concern. The magnitude and frequency of the effect will also be sensitive to additional environmental factors and stressors such as temperature, food availability, predation or habitat loss^18^,^24^,^25^. In addition to this complexity, exposure, biological traits, environmental factors and stressors all vary in space and time. This variability, combined with the uncertainty associated with the quantification of each of the above, result in the probability of an undesired effect. Methods to integrate different levels of uncertainty and variability in probabilistic risk assessment exist^26^,^27^,^28^,^29^. However, a major challenge is the integration of multiple environmental factors, stressors, and stress responses.

As an initial step, it is necessary to develop environmental scenarios with defined input variables and parameters that are a qualitative and quantitative description of the relevant environment for the ERA^30^. According to Rico *et al*.^7^ “unified” environmental scenarios consist of a combination of both biotic and abiotic parameters required to characterise direct and indirect exposure, effects, and recovery of species, therefore integrating both ecological scenarios and exposure scenarios. The concept of exposure scenarios has been integrated in prospective ERAs for more than a decade in the EU^31^ and probabilistic exposure assessment through the use of parameter distributions has been used as a part of chemical risk assessment for more than 20 years^7^,^20^,^21^,^22^,^23^,^32^. Despite this, to our knowledge, so far no regulatory submissions have actually used probabilistic approaches with EUSES (European Union System for the Evaluation of Substances)^21^,^22^. An exposure scenario will predict chemical fate on both spatial and temporal scales by integrating information on chemical use, physical-chemical properties, abiotic factors impacting the exposure, anthropogenic practices, and landscape configuration^7^,^31^,^33^.

Conversely, the concept of ecological scenarios is not yet well defined^7^,^8^. Ecological scenarios should include information on ecosystem structure, intra and inter-species ecological interactions, relevant population level traits, exposure susceptibility, as well as abiotic characteristics that may influence the species responses to stressors, landscape configuration, ecological stressors and spatial and temporal scale^7^.

At which level of biological organization should we integrate? How can we calculate the combined response of a population or community to both a toxicant and a variety of environmental factors and stressors? The organismal response to toxicants clearly depends on environmental factors and stressors, hence it appears obvious to integrate at this level of organization. Furthermore, organism responses can be measured in the laboratory. Therefore we model organism responses and then use the organism level model as a building block in population models to extrapolate to higher levels of organization. Extrapolations to the population level of organisation has already been developed and applied^34^,^35^,^36^ but further work is still needed to extrapolate to higher level of organisation (e.g. community level)^37^,^38^. The organism level model needs to simulate life-history parameters such as survival, growth and reproduction because these determine population dynamics. As growth and reproduction are driven by an organisms’ energy balance, Dynamic Energy Budget models are particularly well suited to integrate toxicant and environmental stressors^39^,^40^,^41^,^42^.

## Theory and Method

The results of such an integrated analysis will inevitably yield complex and multi-scaled results. A remaining challenge is to graphically depict the results in a way that is simple enough to understand, yet that retains enough of the complexity to make an informed decision. In current ERAs, assessors usually compare an exposure level to a no-effect level. In Europe, the Predicted Environmental Concentration/Predicted No-Effect Concentration (PEC/PNEC) ratio is often used. The PEC is often derived from mechanistic fate models and can usually be tailored for local environments. The PNEC is typically derived via the application of an assessment (or uncertainty) factor to the most sensitive species or to a species sensitivity distribution and therefore, does not account for environmental variability. It is also important to note that the usual PEC/PNEC ratio is an indication of an exceeded threshold and not a quantification of a risk. The value of this ratio does not explicitly account for uncertainties or variabilities and tells us very little about the nature or level of effect and the likelihood of an undesired event. Further, the relationship between the PEC/PNEC ratio and the level of effect is unknown^43^,^44^.

**Figure 1:**
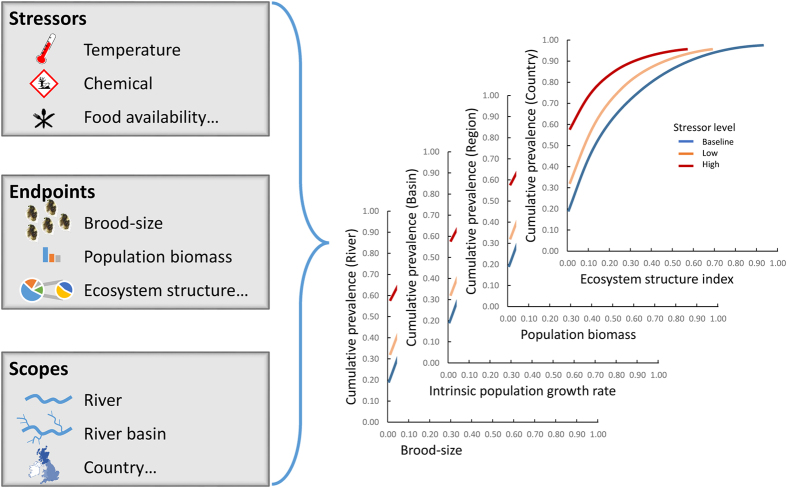
Prevalence plots. The prevalence plot present an endpoint (e.g. brood-size, population biomass; see Fig. 3) or an effect size (e.g. loss of biomass, index relative to the population structure; see Fig. 4) as a function of a cumulative prevalence for this effect (e.g. proportion of a river, hydrogeographic basin) for a selected stress level (e.g. chemical stress, temperature stress). The map was created using GIMP 2.8.14 (www.gimp.org).

The method presented here has the potential to overcome the limitations associated with the PEC/PNEC ratio and similar methods as it is focused on a quantitative measure of effects and it enables the uncertainty and variability in effects and exposure to be quantified and could, ultimately, serve as a replacement. Prevalence plots present an endpoint or an effect size as a function of its cumulative prevalence (Fig. 1). They (i) provide an estimate of risk by using more integrated biological endpoints, (ii) bring greater ecological relevance to risk assessments, and (iii) aid more transparent risk communication by using relevant effect size (e.g. assessor-defined reduction in population biomass) instead of a significant statistical effect (e.g. difference in means of two populations associated with p-values). Prevalence plots can either use “raw” data to represent the effects of the stressor on an endpoint or data scaled by baseline condition to represent the relative impact of the stressor (effect size). Such a plot enables a rapid and meaningful understanding of the effects of stressors and incorporates relevant ecological factors. The endpoints and effect sizes can be tailored depending on one’s needs from rather basic ones such as a brood-size or decrease in population growth rate to more elaborate metrics such as a population biomass or difference in a biodiversity indicator, or even a really complex one such as a score representing a population or ecosystem structure (e.g. Ecological Integrity Assessment^45^,^46^). Likewise, the prevalence axis can be drawn according to one’s needs. It can indeed represent various scales of study such as the prevalence of an effect size in a portion of a freshwater habitat or the prevalence of an effect size in river basins within a region. This type of plot could therefore be used by various communities such as a risk assessor interested in the effect of chemicals discharged on the whole ecosystem of a river or an academic assessing the effect of pollution on a particular species worldwide.

### The Ecological Simulation Model.

To produce a conceptual working example of the prevalence plot, we simulated the response of a population of *Daphnia magna* exposed to stressors using a modified version of the DEB-IBM (Dynamic Energy Budget model coupled with an Individual Based Model) published by Martin *et al*.^47^ to represent the effect size. DEB theory^48^ is based on a mathematical description of the uptake and use of energy within an organism. Energy allocation can be affected by chemicals acting via various physiological modes of action (pMoA)^49^ or by environmental parameters such as temperature or food availability which can affect all the energy fluxes in an organism^48^ (Table 1). The DEB model used in this study is a simplified standard version but it is readily able to relate a stressor level to physiological effects, more complex implementations can handle more subtle effects such as a receptor-mediated effect [ref. 48, see p. 244]. An IBM is used to translate the changes in growth, reproduction, and survival to population endpoints pertinent to support risk assessment such as the population biomass or abundance or the population structure^34^,^50^.

We demonstrate the integration of multiple stressors by varying three conditions in the environment, namely food availability, temperature, and degree of chemical stress. To combine their effects, we modified the DEB part of the DEB-IBM model to account for variability in temperature by adjusting DEB rates according to an Arrhenius relationship


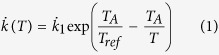


with 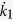 the initial value of the parameter, *T* the actual temperature (K), *T*_*ref*_ the reference temperature (K), and *T*_*A*_ the Arrhenius temperature (K) [ref. 48, see p. 17]. The DEB-IBM model already accounts for food availability^47^ and provides an implementation of equations for modelling chemical stress via different pMoAs^41^. We selected pMoA *maintenance* (i.e. increase of maintenance costs) as our example stressor.

For this exercise, we defined our simulation scenarios with a minimal level of complexity, only considering variations in temperature, food availability, and chemical stress. To illustrate the impact of multiple stressors we chose to simulate the *D. magna* population in two scenarios – one within the temperature tolerance range (Temperate scenario), and another approaching and exceeding the temperature tolerance (Tropical scenario). While not ecologically realistic, this artificial example provides a clear illustrative example of how a chemical stressor can impact the overall performance of populations that are already under pressure from other unrelated stressors. For simplicity, the scenarios are identical with exception of the temperature distribution.

The temperature distributions were based on the temperature recorded in the river Thames from 1974 to 2005 (from 2.0 °C to 26.5 °C) for the *Temperate scenario* and from the river Brahmaputra from 1979 to 1995 (from 19.6 °C to 34.0 °C) for the *Tropical scenario*^55^. The amount of food available in the two scenarios is driven by an arbitrary uniform distribution of the resource dilution rate implemented in the DEB-IBM we used^47^ (from 0.005 to 0.1 by 0.005 increments). To represent chemical exposure and impact on a test species, we applied chemical stress levels ranging from 0 to 1.5 in 0.1 increments. This corresponds to an expected reduction of the reproduction outputs compared to the control of respectively 0% to 95% in the OECD *Daphnia magna* reproduction test (assuming *ad libitum* food at 20 °C) [ref. 47, see SI]. In this illustrative example we have used an arbitrary stress level as described above. In practice, such stress levels can be derived from the predicted environmental concentrations in complex exposure models such as the ScenAT^56^. For further information on extrapolating from an external concentration to the chemical stress level, one can refer to Jager and Zimmer 2012^41^.

The DEB-IBM simulation allowed the *D. magna* population to reach steady-state over the first 150 days of the simulation at which point a constant level of chemical stress was applied until the end of the simulation at 600 days. Outputs were recorded between day 300 and day 600. This allowed sufficient time for the model to reach its new steady-state while avoiding the recording of transient dynamics^47^. Monte-Carlo simulations were used to simulate the *D. magna* population at different combinations of food availability, temperature, and chemical stress level. Each combination consisted of one constant value of food availability, of temperature, and of chemical stress. Thus the simulations do not account for seasonal or other temporal effects. Each combination was simulated three times whilst sampling DEB model parameters to account for inter-individual variability (See Supporting Information). The parameters used for the DEB-IBM simulation are presented in Table S1. It is important to note that we did not account for correlations that may exist between the temperature level and the food availability in these conceptual environmental scenarios. Further, our choice to assess the system at steady-state is appropriate for some questions, but may not be suitable for others where the interest might be in system resilience or structure.

### Visualising the Model Output in Prevalence Plots.

**Figure 2:**
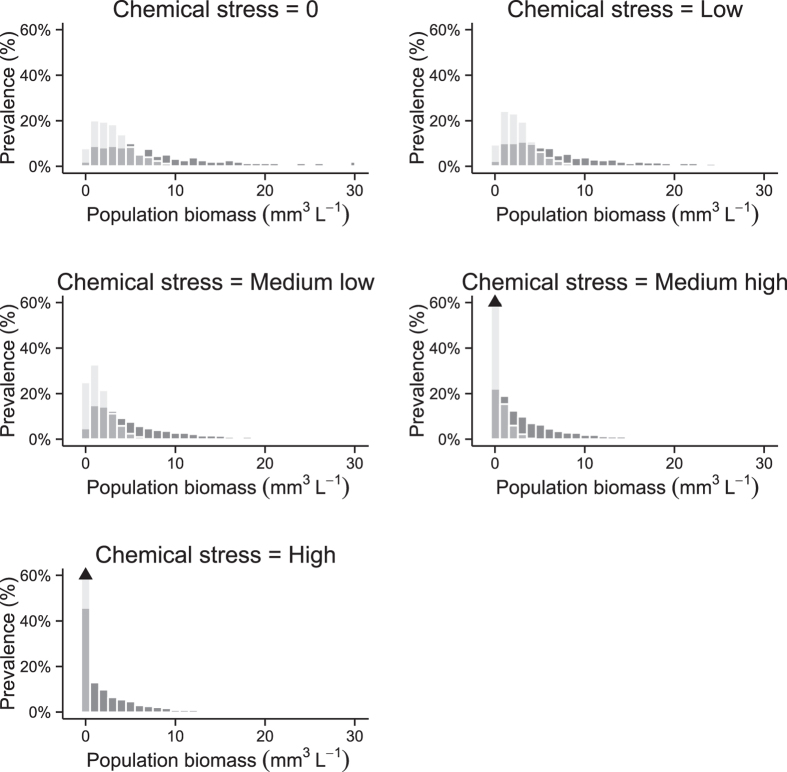
Raw prevalence histogram. Prevalence distribution of the population biomass (mm^3^ L^−1^) for the Temperate (dark grey) and the Tropical (light grey) conceptual scenarios for the five ranges of chemical stress level. The arrows in the “Medium high” and “High” panels denote a prevalence of 76% and 100% respectively.

As an endpoint for our analysis we choose the population biomass calculated as the average sum of the adult individuals’ cubic length over 300 days^47^ relative to the water body volume simulated. In the prevalence plot, an endpoint can be based on the raw value compared to a baseline condition or as an effect size, thus relative to the baseline condition, using for our case study:


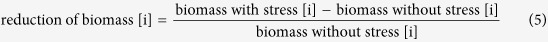


where “i” is the i^th^ combination of environmental parameters. The prevalence plots are based on the frequency distribution of the population biomass for each stress level. This frequency distribution can represent the raw (Fig. 2) or the effect-size data (Fig. S1). The prevalence plots are then constructed by plotting the increasing cumulative sum of the frequency distribution for each range of stress level and for either the raw or the effect-size data. Plotting the cumulative probability on the y-axis is common in probabilistic risk assessment^21^ and environmental fate studies^57^, hence we followed the same convention.

In our study, the cumulative prevalence represents the proportion of all water bodies in a specific location in the Temperate or Tropical scenario having a certain *D. magna* biomass or less. Because our interest is in the contribution of chemical stress to the overall biomass, each plotted line represents a given level of chemical stress. To compare different stressor levels we plot multiple lines. If the question asked concerns one of the other factors, here food availability or temperature, one can plot a line for each level of food availability or each temperature (Fig. 1). In practice it is best to define ranges for the factor being plotted to reduce the number of plotted lines to a manageable number. Thus, we decided to plot the contribution of five ranges of chemical stress (no-chemical 0, low 0.1–0.2, medium low 0.3–0.7, medium high 0.8–1.2, and high 1.3–1.5).

## At the Helm: Decision Making Using Prevalence Plots

In contrast to current ERA, prevalence plots (i) provide an estimate of risk by using more integrated biological endpoints, (ii) bring greater ecological relevance to risk assessments, and (iii) aid more transparent risk communication. We illustrate these three points by constructing prevalence plots for the example of population biomass of *D. magna* exposed to a hypothetical stressor in two illustrative scenarios: Temperate and Tropical.

**Figure 3:**
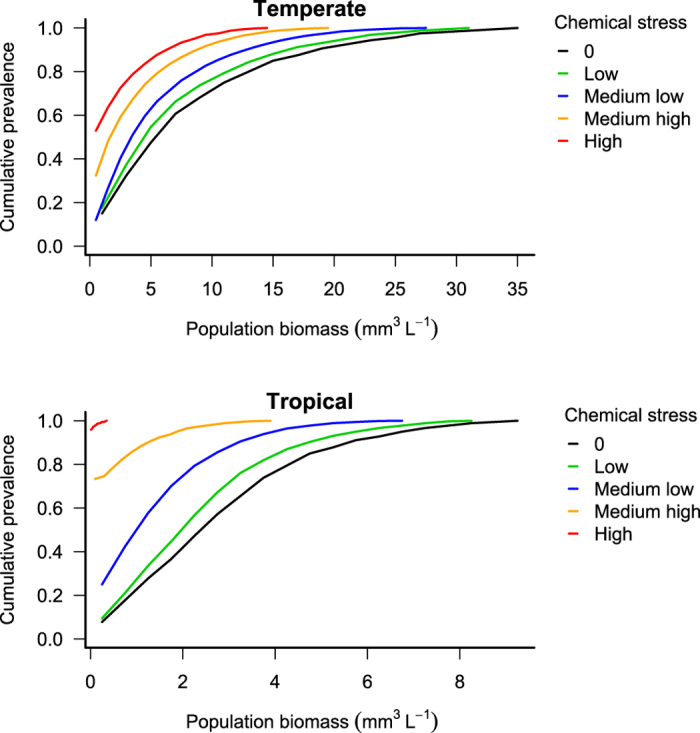
Raw prevalence plot. Population biomass (mm^3^ L^−1^) for the Temperate and Tropical scenarios as a function of the cumulative prevalence for the five ranges of chemical stress.

The raw prevalence distribution (Fig. 2) is the first step before building the raw cumulative prevalence plot (Fig. 3). Figure 2 demonstrates that even without any chemical stress, i.e. the baseline conditions, a difference in the distribution of the population biomasses exists between the Temperate and the Tropical scenarios. The raw prevalence plot (Fig. 3) shows that the baseline population biomass is at maximum 5.50 mm^3^ L^−1^ and 2.37 mm^3^ L^−1^ at the 50^th^ percentile for the Temperate and Tropical scenarios respectively. This denotes the important effect of the environmental scenario on the baseline, or natural, state of ecological endpoints, and therefore, the necessity to carefully build the scenarios. In our illustrative case, the difference between the two scenarios is the temperature distribution, which drives the prevalence distribution of the baseline state of the population biomass.

**Figure 4:**
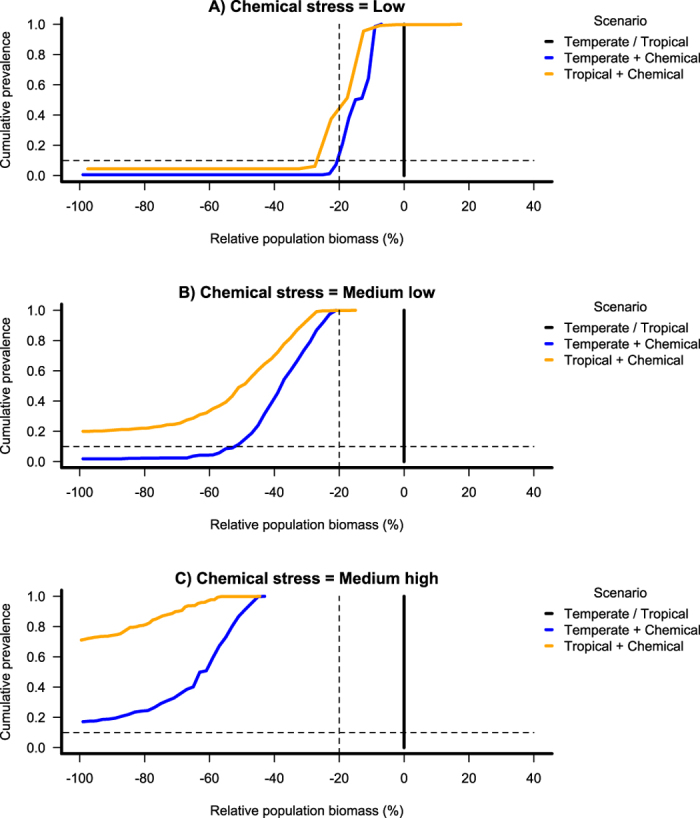
Effect-size prevalence plot. Population biomass relative to the no-chemical stress level population biomass (baseline) as a function of the cumulative prevalence for the baseline state, the Temperate and the Tropical scenarios and for a low, medium low, and medium high level of chemical stress.

While adding a chemical effect, a shift of the distribution towards a lower population biomass is observed in both scenarios (Figs 2 and 3). For example, with a medium low level of chemical stress, the population biomass would be at maximum 3.31 mm^3^ L^−1^ and 1.07 mm^3^ L^−1^ at the 50^th^ percentile in the Temperate and Tropical scenarios respectively. As we are interested in the effects of the chemical stress on the population biomass, we can also plot effect-size prevalence plots (Figs 4 and S2) based on the effect-size prevalence distribution (Fig. S1). Such plots represent the population biomass relative to a baseline state as a function of the cumulative prevalence. This baseline state includes the effects of natural stressors but not the effects of the stressor of interest (chemical stress in our illustration). As the effect-size plot compares the predicted state to a baseline state, the baseline, “no chemical” line is the same for both the Temperate and the Tropical scenario. Figure 4A shows that the population biomass of individuals exposed to a low level of chemical stress is reduced by at least ca. 15% compared to the population biomass of the non-exposed individuals (baseline state) in 50% of the cases for the Temperate scenario. This reduction would be at least ca. 18% for the Tropical scenario. While increasing the chemical stress level (Fig. 4B,C), the impact on the population biomass increases in both scenarios, but more dramatically in the Tropical scenario. For example, with a medium high level of chemical stress (Fig. 4C), in 50% of the cases, the population biomass would be reduced by at least ca. 60% and 100% in the Temperate and Tropical scenarios, respectively. This denotes a higher risk of population extinction in the latter conceptual scenario for that specific species.

It is ultimately a policy decision to define a maximal prevalence (e.g. the proportion of rivers) and impact (e.g. percent reduction in biomass) that are ecologically acceptable for different systems. For instance, a protection goal where 90% of rivers have less than 20% reduction in river biomass due to additional chemical stress could be deemed acceptable compared to a baseline scenario (dashed line on Fig. 4). In such a case, our Temperate scenario would indicate that a low level of chemical stress is acceptable but our Tropical scenario would not be acceptable at this level of chemical stress (Figs 4 and S2). This policy decision could be tailored to ecological, local, commercial or political needs. For endangered, key ecosystem or key touristic or commercial species for example, the policy decision could be to allow no more than 95% of river systems to see a 5% reduction in population biomass whereas in areas set aside for intensive agricultural production a stronger impact might be considered acceptable. Whatever the policy decision is, it can be translated into maximum acceptable chemical stress levels using prevalence plots, whilst accounting for ecological factors and variable environmental conditions.

## Back to the Engine Room: Improving the Underlying Science

In order to obtain meaningful and non-biased results, it is necessary to carefully build the unified environmental scenarios. In our illustrative example, both the Temperate and the Tropical scenarios are based on a single species, *D. magna*. Yet, our Tropical scenario fell outside of *D. magna* temperature tolerance range. Thus, the individuals are already stressed by a non-optimal temperature environment which can induce a lower capability of the population to cope with a new stress^58^. Therefore, the addition of another stress (here a chemical stress) induces a higher adverse effect on the population biomass. The selection of a relevant species is thus an important step of the construction of the environmental scenario. Indeed, it has been demonstrated that, even if some individual differences exist for specific compounds and taxa^59^,^60^, no clear pattern in differences in sensitivity exist between tropical and temperate species exposed to chemical stress under laboratory or semi-field/mesocosm conditions^59^,^60^,^61^. Kwok *et al*.^59^ for instance, assessed the differences in species sensitivity between temperate and tropical species for 18 chemicals using species sensitivity distributions (SSD) comparisons. The authors found that no real pattern exists between the species from the two different climatic zones but noted that temperate species seem more sensitive to metals. Conversely, they observed the reverse result for other types of chemicals such as chlorpyrifos, un-ionized ammonia or phenol. Similarly, no real pattern was found by Daam and van den Brink^61^ who assessed the differences between temperate and tropical freshwater ecosystems for the ERA of pesticides. They concluded that vulnerability of freshwaters to pesticides did not consistently differ between tropical and temperate ecosystems. However, it is important to note that these studies are conducted under laboratory or relatively clean semi-field “pristine” conditions and do not reflect the variability of real world multi-stressed environments. Indeed, the impact of multiple stressors on aquatic communities is an active and growing area of research^10^,^18^,^62^,^63^. Characterisation of these stressors in diverse scenarios and their influence on the aquatic community composition and chemical fate and behaviour is critical to better understand potential differences in chemical stress on Temperate and Tropical scenarios.

Currently our illustrative scenario only includes three stress factors (food availability, temperature, and chemical) and a simple question about the effect of chemical stress at steady state. But with further development other factors (Table 1) and questions can also be tackled with the framework we present. Indeed, one can be interested in the effect of stressors on dynamic transition states of a population in order to assess the resilience capabilities of this population. One can also be interested in a scenario accounting for the seasonal dynamics of food or photo period (e.g. for algae) or even for more complex ecological interactions such as the impact of competition and/or predation on the response of a population to a chemical stressor or the impact of a reduced dissolved oxygen in areas receiving untreated wastewater^64^.

Whereas prevalence plots presented here only account for the unified environmental scenario variability, it is possible to include the analysis of the uncertainty as well in to these plots (Fig. S3). One can calculate the uncertainty around the effect size using a bootstrap method for example. These uncertainties exist in all levels of the scenarios. Thus, it may exist on the baseline state, based on the uncertainties around abiotic factors such as the temperature, pH, dissolved oxygen, water quality measurements and biotic factors such as the species selection, the ecological processes to include in the scenarios, or even the quality of the biomonitoring data. Uncertainties will also be related to the fate of the stressor of interest with factors of uncertainty such as a chemical emission and use or degradation behaviour. It may also exist as the intrinsic uncertainty of the model equations and of the model calibration processes. The uncertainty is assumed to be reduced according to the refinement used while building the unified environmental scenario. Thus, lower tier ERA, that may include more generic scenarios, will lead to a higher uncertainty whereas higher tier ERA, with refined and specific scenarios and more data, will lead to a lower and explicitly characterised uncertainty.

**Figure 5:**
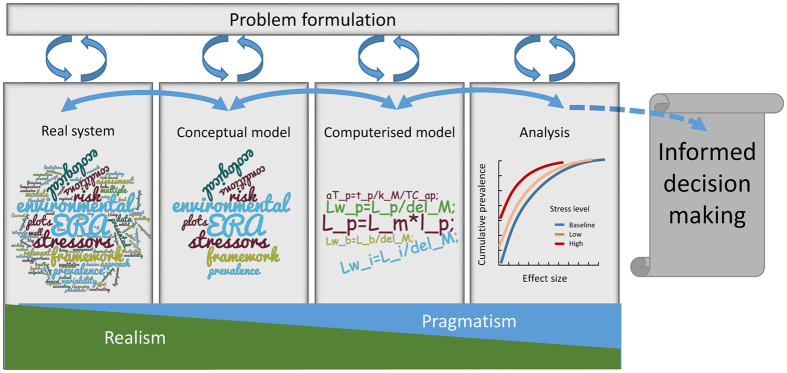
Schematic view of the framework and the underlying scientific improvements needed.

Although, in principle, this framework could be used by regulators and decision makers, it requires additional scientific research to improve various components (Fig. 5). The scope of this iterative framework will increase with scientific and technological advances. For example, as we continue to better understand the real systems our ability to characterise key ecosystem functioning (e.g. characterisation of food webs, ecosystem resilience) will aid the refinement of the problem formulation and the development or update of conceptual models. The creation of the latter is indeed, as all parts of the framework, an iterative process that will require fit for purpose environmental scenarios that are adequately characterised in terms of key ecosystem components, selection of key species, ecological interactions, key abiotic factors and chemical fate and behaviour and that can be improved with increasing knowledge. The translation of these components and their interactions into a mathematical and computerised models also requires scientific improvements, especially in modelling theories able to cope with multiple species, multiple interactions, and multiple stressors. Finally, the analysis of the outputs of such models requires prior knowledge and accurate definition of the protection goals. However, what we present here already are meaningful metrics and a means of communicating potential risks of chemicals to the environment for a range of stakeholders.

## Conclusions

It is widely acknowledged that current environmental risk assessment methodologies for single stressors are conservative and generally protective. However, in order to incorporate advances in scientific understanding, improve ecological realism (multiple stressors) and better account for uncertainty and variability a new framework is required. We presented a novel framework that enables ERA to use unified environmental scenarios that combine mechanistic effects models with exposure models to overcome these limitations. The framework can already be used to aid decision making, but it does require a good understanding of key ecological services that are of specific interest to risk assessors in order to build relevant unified environmental scenarios. Another priority to improve the framework is to better account for multiple stressors in ERA and improve our ability to extrapolate effects to ecologically relevant levels of organisation. Interactions of chemicals with each other and with environmental factors can modulate bioavailability, toxicokinetics (TK, uptake, distribution, biotransformation, and depuration), toxicodynamics (TD, action on the biological target) or both TK and TD^65^. Whereas ideas and equations exist for the integration of single toxic and ecological stressors (see Table 1 for a vision on the integration of single stressors in DEB models), a knowledge gap still exists on the integration of the effects of combined stressors (both chemical and ecological). Such combined effects on individuals could be taken into account by impacting the relevant energy fluxes in the DEB framework. This, however, requires caution as the type, the sign, and the strength of the effect seems to be dependent on multiple factors (such as the prey, parasite, or predator type, the abundance of each species, the type of habitat, etc.) and the consequences of their interactions for an organism’s energy budget are poorly understood. This framework is computationally intensive, especially for high resolution, spatially explicit environmental scenarios therefore optimising the framework as well as the model parametrisation and model analysis are needed to implement this framework efficiently.

Stakeholders and decision makers can begin to use the framework. Importantly, an iterative process needs to be initiated early with input from risk assessors and risk managers to better inform model developers so that the framework can be optimised to ensure the right balance between realism and pragmatism is incorporated and that the output enables a shift from threshold ERA, driven by summary statistics such as the PEC/PNEC ratio, to a more mechanistic and risk based ERA. Indeed, the use of prevalence plots provides a more transparent, quantitative and meaningful interpretation of environmental risk than conventional PEC/PNEC threshold approaches. The prevalence plots represent a flexible interface between the risk assessment and the risk management, which explicitly accounts for the effect of complex interactions as well as variabilities and uncertainties that can be tailored to bespoke problem formulations.

## Additional Information

**How to cite this article**: Goussen, B. *et al*. Integrated presentation of ecological risk from multiple stressors. *Sci. Rep.* **6**, 36004; doi: 10.1038/srep36004 (2016).

**Publisher’s note:** Springer Nature remains neutral with regard to jurisdictional claims in published maps and institutional affiliations.

## Supplementary Material

Supplementary Information

## Figures and Tables

**Table 1:** Examples of integration rules.

Environmental factor	DEB fluxes impacted	Relationship	Reference
Predation	Indirect effect: assimilation and/or reproduction	Indirect effect: likely to be reduction of assimilation and/or direct effect on reproduction triggered by predator cues	Based on^16^,^51^,^52^,^53^
Parasitism	Variable	Highly dependent on the parasite and the species	Based on^18^,^54^
Resources competition	Assimilation	Decrease of the assimilation fluxes (likely to be due to a reduction of the resources availability)	
Temperature	All rates ( 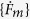 , 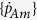 , 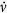 , 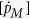 , 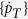 , 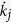 )	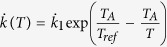 (2) All rates should depend on temperature in the same way in the standard DEB model	48
Food availability	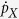 (ingestion rate)	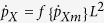 with 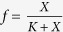 (3)	48
	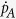 (assimilation rate)	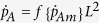 with 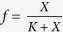 (4)	48
Oxygen deficit	All rates	Could be accounted for as a specific substrate with its own sets of parameters (which can be partly identical to the parameters of the other substrates)	
Toxic compound	Depending on the pMoA*	Impact on energetic fluxes depending on pMoA	41
Other ecological stress	Variable	Depends on the behaviour modification	

Illustrations of the possible impacts of environmental parameters on the energy fluxes of the Dynamic Energy Budget theory.

^*^Five physiological modes of action (pMoA) are commonly described in the DEB literature, namely a decrease of the *assimilation* of energy from food, an increase of the *maintenance* costs, an increase of the cost to create an unit of structure (*cost for growth*), an increase of the *cost for creating an egg*, or an *hazard during the oogenesis* process^41^. With 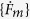 the specific searching rate, 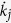 the maturity maintenance rate coefficient, 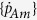 the surface-area-specific maximum assimilation rate, 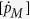 the specific volume-linked somatic maintenance rate, 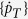 the specific surface-area-linked somatic maintenance rate, 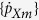 the surface specific maximum ingestion rate, 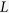 the structural length of the individual, 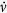 the energy conductance, 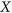 the food density, *K* the half-saturation coefficient, 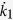 the initial value of the parameter, *T* the actual temperature, *T*_*ref*_ the reference temperature, and *T*_*A*_ the Arrhenius temperature.
