# Supplementary material for: Integrated presentation of ecological risk from multiple stressors
Source: Sci Rep. 2016 Oct 26;6:36004. doi: 10.1038/srep36004 (PMC5080554; doi:10.1038/srep36004)
Supplement: Supplementary Information [file srep36004-s1.pdf]

Integrated presentation of ecological risk from multiple stressors

Benoit Goussen[1,2,*], Oliver R. Price[2], Cecilie Rendal[2], and Roman Ashauer[1]

[1]Environment Department, University of York, Heslington, York, YO10 5DD, United Kingdom

[2]Safety and Environmental Assurance Centre, Colworth Science Park, Unilever, Sharnbrook, Bedfordshire, United Kingdom

[*]benoit.goussen@gmail.com

Supporting information

Inter-individual variability

The inter-individual variability was achieved according to the method describe by Kooijman et. al.[1] To do so, each combination was simulated three times whilst sampling DEB model parameters by multiplying the input DEB parameters linked to the surface-area-specific maximum assimilation rate of an individual by a scatter-multiplier. The multiplier was derived from a log-normal distribution with standard deviation of 0.05.[1,2]

Monte-Carlo simulations

We used Monte-Carlo simulations to sample parameters values in the Chemical stress level, Temperature, and Food distributions (Chemical stress level $\times$ Temperature stress $\times$ Food stress $\times$ Stochasticity). We performed 7680 runs for the Temperate scenario ($16 \times 8 \times 20 \times 3$) and 8640 runs for the Tropical scenario ($16 \times 9 \times 20 \times 3$) which allow us to cover efficiently the distribution of the selected parameters.

Table S1. DEB-IBM parameters values.

Parameter	Description	Value
κ	Fraction of mobilised energy to soma (-)	0.678[3]
κ_R	Fraction of reproduction energy fixed in eggs (-)	0.95[3]
$\dot{k}_m$	Somatic maintenance rate coefficient (d^{-1})	0.3314[3]
$\dot{k}_j$	Maturity maintenance rate coefficient (d^{-1})	0.1921[3]
U_H^b	Scaled maturity at birth ($d\,mm^2$)	0.1108[3]
U_H^p	Scaled maturity at puberty ($d\,mm^2$)	2.547[4]
$\dot{v}$	Energy conductance ($mm\,d^{-1}$)	18.1[3]
g	Energy investment ratio (-)	10[3]
$\ddot{h}_a$	Hazard rate - ageing parameter (d^{-2})	3.04×10^{-6},[3]
s_G	Gompertz stress coefficient (-)	0.019[3]
cv	Coefficient of variation of the parameter values (-)	0.05[3]
$\{\dot{J}_{XAm}\}$	Maximum specific ingestion rate (algae-cells $mm^{-2}\,d^{-1}$)	380 000[3]
H	Half-saturation coefficient (algae-cells cm^{-3})	1585[3]
$juv\text{-}mort$	Juvenile resource-dependant mortality rate (d^{-1})	0.09[3]
$t\text{-}molt$	Days between reproduction molts (d)	2.8[3]
ρ	Resource dilution rate (d^{-1})	$U\,[0.005, 0.1]$
R_{Max}	Maximum resource density (algae-cells cm^{-3})	7925[4]
$Volume$	Volume of the water simulated (cm^3)	200 000
$Stress\text{-}level$	Level of chemical stress (-)	$U\,[0, 1.5]$ [4, Based on]
T	Temperature of the environment (°C)	Based on rivers Thames and Brahmaputra[5]
T_A	Arrhenius temperature (K)	6400[6]
T_{ref}	Reference temperature (K)	293[6]

The uniform distribution between a and b is noted $U\,[a, b]$.

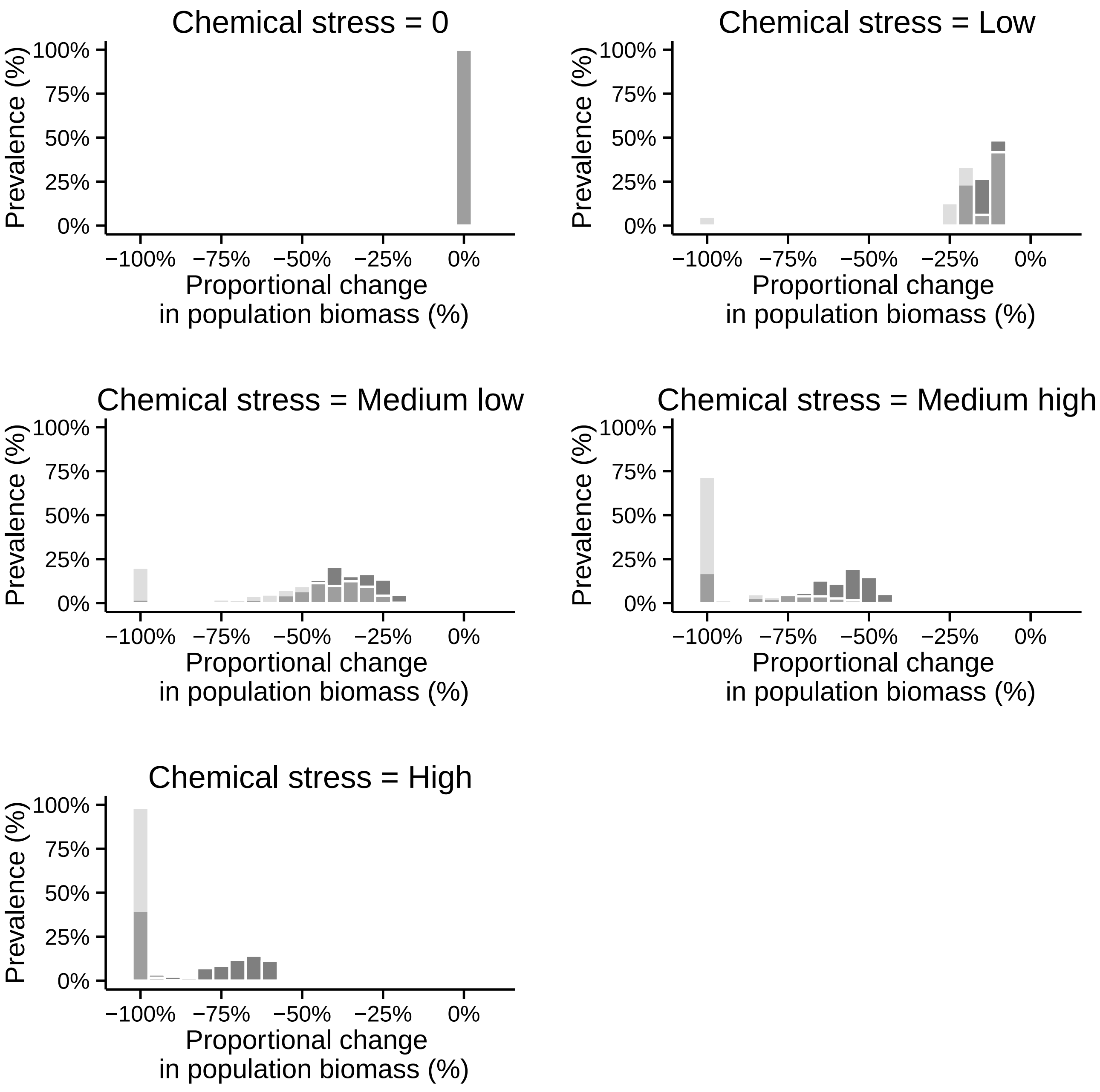

Figure S1. Effect-size prevalence histogram. Prevalence distribution of the relative population biomass for the Temperate (dark grey) and the Tropical (light grey) conceptual scenarios for the four ranges of chemical stress level.

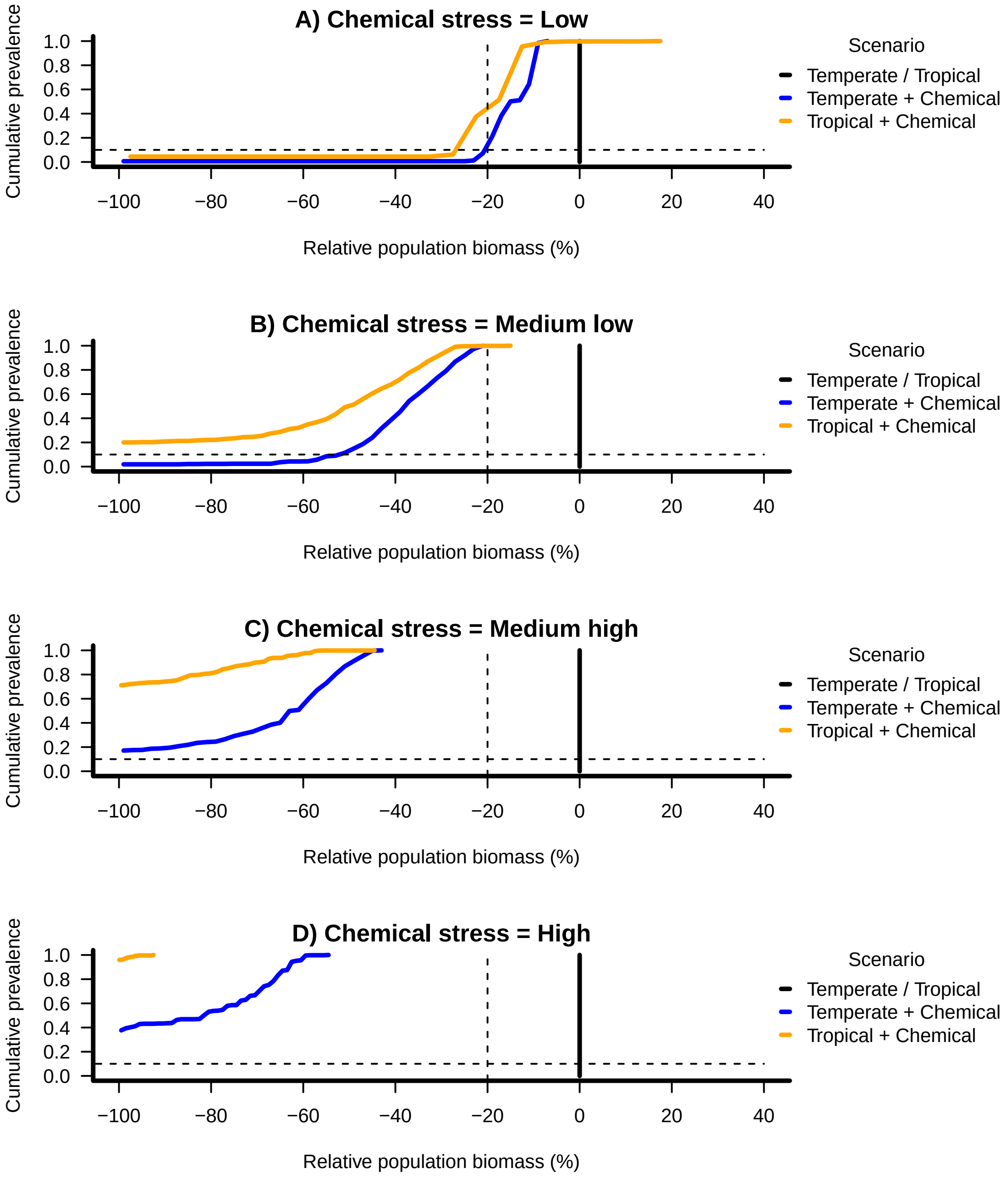

Figure S2. Effect-size prevalence plot. Population biomass relative to the no-chemical stress level population biomass (baseline) as a function of the cumulative prevalence for the baseline state, the Temperate and the Tropical scenarios and for a low, medium low, medium high, and high level of chemical stress.

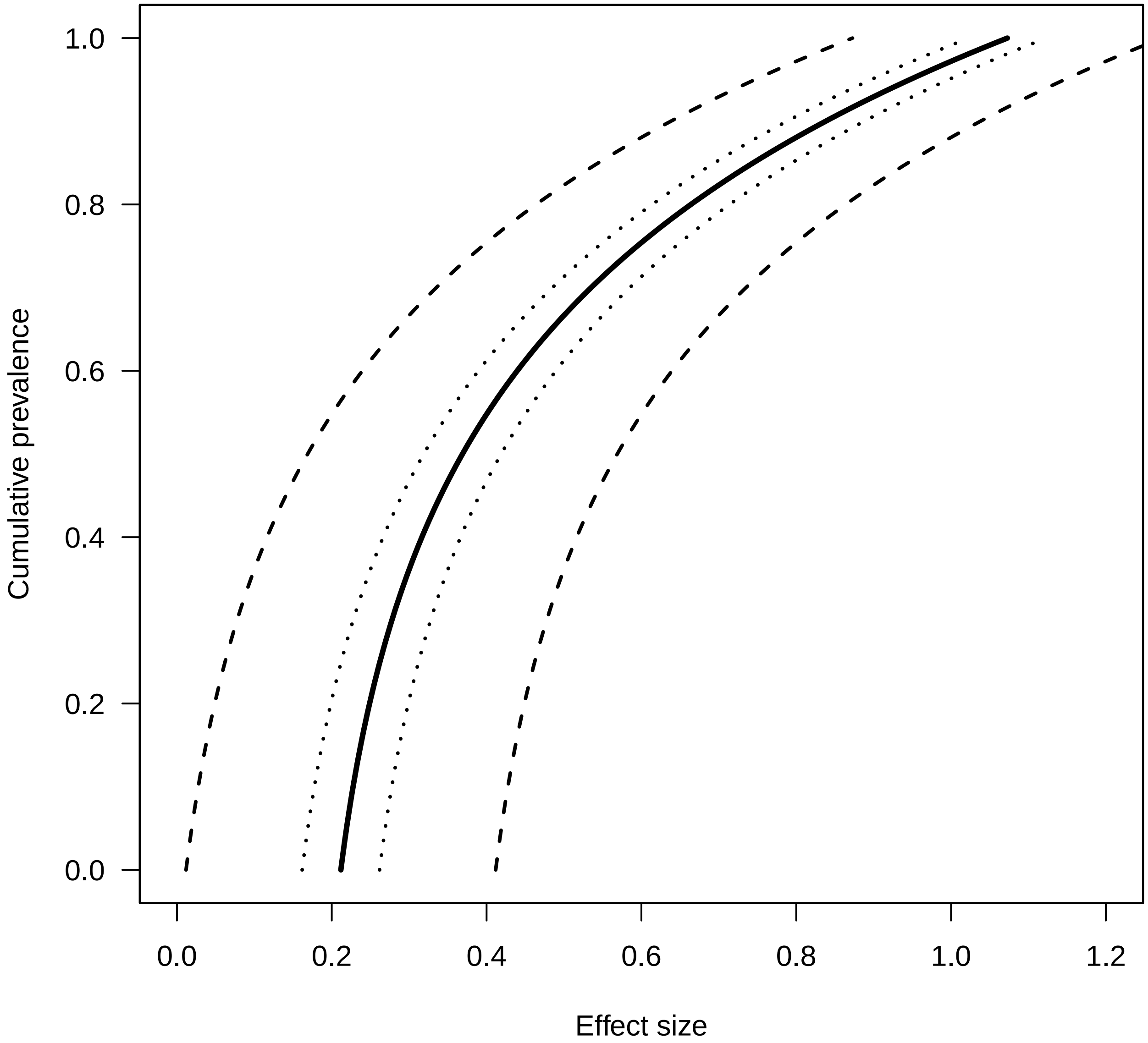

Figure S3. Prevalence plot with uncertainties. Representation of an effect size as a function of its cumulative prevalence (*solid line*) with a low tier (*dashed lines*) and a high tier (*dotted lines*) uncertainty analysis.

References

1. Kooijman, S. A. L. M., Van Der Hoeven, N. & Van Der Werf, D. C. Population consequences of a physiological model for individuals. *Funct. Ecol.* **3**, 325–336 (1989). URL http://www.jstor.org/stable/2389373.

2. Martin, B. T., Zimmer, E. I., Grimm, V. & Jager, T. Dynamic Energy Budget theory meets individual-based modelling: a generic and accessible implementation. *Methods Ecol. Evol.* **3**, 445–449 (2012).

3. Martin, B. T., Jager, T., Nisbet, R. M., Preuss, T. G. & Grimm, V. Predicting Population Dynamics from the Properties of Individuals: A Cross-Level Test of Dynamic Energy Budget Theory. *Am. Nat.* **181**, 506–519 (2013).

4. Martin, B. T., Jager, T., Nisbet, R. M., Preuss, T. G. & Grimm, V. Limitations of extrapolating toxic effects on reproduction to the population level. *Ecol. Appl.* **24**, 1972–1983 (2014).

5. GEMStat. United nations environment programme global environment monitoring system water programme (2004). GEMStat © 2004.

6. Kooijman, S. A. L. M. *Dynamic Energy Budget Theory for Metabolic Organisation* (Cambridge University Press, Cambridge, 2010), third edn.
